# Every road leads to Rome: therapeutic effect and mechanism of the extracellular vesicles of human embryonic stem cell-derived immune and matrix regulatory cells administered to mouse models of pulmonary fibrosis through different routes

**DOI:** 10.1186/s13287-022-02839-7

**Published:** 2022-04-12

**Authors:** Shengnan Yang, Peipei Liu, Tingting Gao, Dingyun Song, Xinyu Zhao, Yupeng Li, Jun Wu, Liu Wang, Zai Wang, Jie Hao, Chen Wang, Huaping Dai

**Affiliations:** 1 Harbin Medical University, Harbin, 150081 Heilongjiang Province China; 2 Department of Pulmonary and Critical Care Medicine, Center of Respiratory Medicine, China-Japan Friendship Hospital, Beijing, 100029 China; 3 National Center for Respiratory Medicine, Beijing, 100029 China; 4 Institute of Respiratory Medicine, Chinese Academy of Medical Sciences, Beijing, 100029 China; 5 Chinese Academy of Medical Sciences, Peking Union Medical College, Beijing, 100730 China; 6 National Stem Cell Resource Center, Chinese Academy of Sciences, Beijing, 100190 China; 7 State Key Laboratory of Stem Cell and Reproductive Biology, Institute of Zoology, Chinese Academy of Sciences, Beijing, 100101 China; 8 Institute for Stem Cell and Regeneration, Chinese Academy of Sciences, Beijing, 100101 China; 9 Beijing Institute for Stem Cell and Regenerative Medicine, Beijing, 100101 China; 10 University of Chinese Academy of Sciences, Beijing, 100049 China; 11 Institute of Clinical Medical Sciences, China-Japan Friendship Hospital, Beijing, 100029 China

**Keywords:** IMRC, Human embryonic stem cells, Mesenchymal stem cells, Pulmonary fibrosis, Extracellular vesicles, Biodistribution, Route of administration

## Abstract

**Background:**

Idiopathic pulmonary fibrosis (IPF) is a progressive and fatal interstitial lung disease. Whether extracellular vesicles are effective in treating IPF and what is the optimal administrative route is not clear. Our previous studies have shown that immunity and matrix regulatory cells (IMRCs) derived from human embryonic stem cells can safely treat lung injury and fibrosis in mouse models, and its mechanism of action is related to the paracrine effect. In this study, we investigated the therapeutic effects of IMRC-derived extracellular vesicles (IMRC-EVs) on a bleomycin-induced pulmonary fibrosis mouse model and explored the optimal route of administration.

**Methods:**

To study the biodistribution of IMRC-EVs after administration via different routes, NIR labeled-IMRC-EVs were delivered by intratracheal (IT) or intravenous (IV) route, and in vivo imaging was acquired at different time points. The therapeutic effects of IMRC-EVs delivered by different routes were analyzed by assessing histology, lung function, cytokines levels, and transcriptome profiling. RNA-seq of lung tissues was performed to investigate the mechanisms of EV treatment through IT or IV administrations.

**Results:**

IMRC-EVs mainly reserved in the liver and spleen when administrated via IV route; and mainly retained in the lungs via the IT route. IMRC-EVs administrated via both routes demonstrated a therapeutic effect as attenuated pulmonary fibrosis, improved lung function, and histological parameters. Based on our RNA-seq results, different pathways may be affected by IMRC-EVs administrated via IT or IV routes. In addition, in vitro experiments showed that IMRC-EVs inhibited epithelial-to-mesenchymal transition induced by TGF-β.

**Conclusion:**

IMRC-EVs administrated via IT or IV routes generate different biodistributions, but are both effective for the treatment of bleomycin-induced pulmonary fibrosis. The therapeutic mechanisms of IMRC-EVs administrated via different routes may be different.

**Supplementary Information:**

The online version contains supplementary material available at 10.1186/s13287-022-02839-7.

## Background

Idiopathic pulmonary fibrosis (IPF) is a chronic and irreversible interstitial lung disease with various causes such as smoking, aging, environmental factors, viral infections, radiation, drugs, and epigenetic/genetic factors [1–3]. The prognosis of the disease is poor. The average survival time of patients over 65 years after diagnosis is 3.8 years [4], and the mortality rate within 5 years after diagnosis is as high as 40% [5], which is even more than that of numerous malignant tumors [6]. However, limited treatment options are available. Apart from lung transplantation, nintedanib and pirfenidone are the only two approved treatments for IPF, but only slow down the decline of lung function. Therefore, there is an urgent need to identify novel therapies to treat this complex disease.

Mesenchymal stem cells (MSCs) are multipotent cells that have immunomodulatory and tissue repair properties [7], which make them ideal candidates for cell therapy of IPF [8]. The homogeneity of cell quality is crucial for the stable therapeutic effect, which was difficult to achieve by the traditional method of MSCs production [9, 10]. Recently, various protocols have been used to generate mesenchymal stem cell-like cells from human embryonic stem cells (hESCs) to improve cell homogeneity and therapeutic effects [11]. We have demonstrated that our hESC-derived MSC-like immune and matrix regulatory cells (IMRC) can improve the survival rate and lung function and reduce pulmonary fibrosis induced by bleomycin (BLM) in a mouse model. In vivo tracking technology and immunofluorescence co-staining showed that IMRCs do not exhibit long-term implantation and direct differentiation, and the main action mechanism of IMRCs is based on paracrine functions [12].

Extracellular vesicle (EV) is a crucial part of the secretome. It plays an important role in MSCs-mediated tissue repair by mediating the intercellular communication between MSCs and the damaged tissues and organs [13]. EVs isolated from the conditioned medium of stem cells have similar effects as parent cells [14, 15]. MSC-derived EVs (MSC-EVs) can replace MSCs for cell therapy to reduce lung damage and promote lung repair [16]. Compared with MSCs, MSC-EVs as acellular therapeutic agents has more obvious advantages for lung diseases, including no self-replication ability, reducing the risk of iatrogenic tumor formation, easily penetrating through biological barriers, and reducing the risks of pulmonary microemboli. In addition, EVs have “off-the-shelf” advantages of easy storage and immediate availability. Therefore, MSC-EVs are considered to be promising therapeutics for pulmonary diseases.

Understanding the optimal delivery route for EV to treat IPF is an important step in translating preclinical findings into the clinical setting. In our previous study, we found that infusion of IMRCs through the tail vein generates obvious therapeutic effect in a mouse model of pulmonary fibrosis, but it is not clear whether the effective route of EVs infusion is the same as that of the parental cells. Since intratracheal (IT) or intravenous (IV) routes have been widely used for drug delivery to treat pulmonary diseases, we compared the biological distributions, therapeutic effects and potential mechanisms of EV treatment through these two routes in the pulmonary fibrosis mouse models. We found that both IT or IV routes have therapeutic effects, but different delivery routes can generate different biological distributions and have different mechanisms of action.

## Methods

### Generation and characterization of hESC-derived IMRCs

Human embryonic stem cell-derived IMRCs were generated from an hESC cell line (Q-CTS-hESC-2) in a serum-free medium according to our previous study [12]. Cells were passaged when they reached approximately 80% confluence in the IMRC culture medium. After culturing for five generations, the differentiated culture was harvested for EV purification.

### Cell culture

MRC-5 cells (CCL-171TM, ATCC) were purchased from ATCC. To prepare the conditioned medium (CM), the EVs in the serum (FBS10091148, Gibco, USA) were first excluded by ultracentrifugation. MRC-5 cells were cultivated in α-MEM medium (01-042-1ACS, Biological Industries, Israel) supplemented with 10% FBS. The cell culture supernatant was harvested when the cells reached 80% confluence. A549 cells (CCL-185, ATCC) were purchased from ATCC. Cells were cultivated in Dulbecco’s modified Eagle’s medium (DMEM) (06-1055-57-1ACS-1, Biological Industries, Israel) containing 10% FBS. For the induction of epithelial-to-mesenchymal transition (EMT), A549 cells were seeded into 6-well plates at a density of 1 × 10^5^ cells/well. Subsequently, cells were treated with 2 ng/ml TGF-β1 (100–21 PeproTech Inc, USA) with or without 100 μg IMRC-EVs for 48 h.

### Separation of EVs by differential ultracentrifugation

To harvest the CM, centrifugation was first performed at 300 and 2000*g* (10 min each) to remove cells, and then at 10,000*g* for 30 min to remove cell debris. The sample was then filtered through a 0.22-μm filter (Millipore, USA) to remove any larger particles. The cell-free supernatant was ultracentrifuged at 110,000*g* for 90 min to precipitate EV. The second washing step was performed by resuspending the EV pellets in 25 mL of phosphate-buffered saline (PBS) and ultracentrifuging at 110,000*g* for 90 min. Then, the EV pellets were resuspended in PBS and stored at − 80 °C. All ultracentrifugation steps were performed using ultracentrifuges (Optima™XPN, Beckman Coulter, Inc., USA) SW32Ti and SW41Ti rotors at 4 °C [17].

### Characterization of IMRC- and MRC-5 derived EVs

EVs were characterized according to the standards recommended by the ISEV position paper [18]. First, transmission electron microscope (TEM) was used to examine the morphology of EVs. EVs (10 µl) were applied to the copper grid for 10 min. After washed once with PBS removal of excess liquid with filter paper and dried naturally, the grid was stained with 2% uranyl acetate for 1 min. The copper grids were dried naturally and observed under TEM (Hitachi H-7650, Japan). Meanwhile, we analyzed the size and number of EVs by nanoparticle tracking analysis (NTA). ZetaView PMX 110 (Particle Metrix, Meerbusch, Germany) and the corresponding software ZetaView 8.04.02 were used for NTA. In addition, the protein content of the concentrated EVs was determined using the bicinchoninic acid (BCA) protein assay kit (Solarbio, China). Successful EVs isolation was confirmed by Western blot analysis for positive EV markers (CD63, CD81 and TSG101) and a negative marker calnexin (Abcam, USA).

### Animal procedures

Specific pathogen-free C57BL/6 N male mice (8–10 weeks old) were purchased from Vital River Laboratory Animal Technology Co., Ltd, Beijing, China. To study the liver and kidney toxicity of the IMRC-EVs in mice, the mice were randomly assigned to three groups (n = 3 or 4 per group) to receive a single intravenous injection of either normal saline or two different dosages (200 μg or 1000 μg) of IMRC-EVs. The mice were euthanized 5 days after the injection and the serum was collected for biochemical analysis. The serum levels of glucose, urea, creatinine (CREA), aspartate aminotransferase (AST), alanine aminotransferase (ALT), total bilirubin, triglyceride, total protein, lactic dehydrogenase (LDH), creatine kinase (CK), albumin and alkaline phosphatase were determined to evaluate the glucose metabolism and kidney and liver damage, using a Hitachi 7600 automatic analyzer (Hitachi, Tokyo, Japan).

To study the influence of cell origins, the mice were randomly divided into four groups (six mice/group): Control group, BLM group, BLM + IMRC-EVs group and BLM + MRC-5-EVs group. The control group received an injection of 50 μl normal saline through laryngoscopy, and the BLM model group (BLM) received 2.5 U/kg bleomycin hydrochloride (BLM; H20055883, Hisun Pharmaceutical Co. Ltd., China) through single intratracheal injection. In the BLM + IMRC-EVs or BLM + MRC-5-EVs group, IMRC-EVs (200 μg/mouse) or MRC-5-EVs (200 μg/mouse) were injected intravenously on the first and second day after BLM injury, respectively.

To study the influence of delivery route of IMRC-EVs, the mice were divided into four groups (each containing 10 mice): Control group, BLM model group, tracheal route (IT-EVs) group, and tail vein route (IV-EVs) group. In the IT-EVs or IV-EVs group, IMRC-EVs (200 μg/mouse) were administered through the trachea or tail vein after BLM injury, respectively. The mice were euthanized on the 21st day after BLM injury. The lung tissues were collected for RNA, protein and histological analysis.

### Physiological measurements

After administration of BLM, weight changes and survival rates were monitored daily. The body weight and lung weight of mice were measured and recorded. Lung coefficient was calculated by the following equation: lung wet weight (g)/body weight (kg) × 100%.

### Histological staining and immunostaining analysis

Hematoxylin and eosin (H&E) staining, Masson staining, and Sirius red staining were used for histological analysis [12, 19]. Anti-α-SMA (Abcam, USA) and type I collagen (Abcam, USA) antibodies were used for immunohistochemical staining. Representative images were acquired with a light microscope (DM4000B, Leica, Wetzlar, Germany). Image analysis was conducted with Image Pro-Plus software.

### Hydroxyproline determination

The hydroxyproline (HYP) level in the lung tissue was measured by using a commercial kit (NBP2-59747, Novus Biologicals, USA) according to the manufacturer’s protocol. The absorbance of the sample was measured at 560 nm and converted to the HYP concentration (μg/mg).

### Detection of IMRC-EVs incorporation

The lipophilic dye PKH-26 was used to label the IMRC-EVs [20]. To test whether IMRC-EVs can be taken up by alveolar epithelial cells in vitro, IMRC-EVs were labeled using PKH26 Red Fluorescent Cell Linker Kits (PKH26GL, Sigma-Aldrich, USA) were co-cultured with the target cells for 3 h. To confirm the internalization of EVs, the cellular actin cytoskeleton was visualized with phalloidin-FITC (40735ES75, Yeasen, China). The nuclei were stained with DAPI. Images were acquired using a fluorescence confocal microscope (LSM800, Zeiss, Germany).

### Immunofluorescence staining

TGF-β-treated A549 cells were cocultured with IMRC-EVs for 48 h before analysis. The cells were rinsed, fixed and permeabilized prior to staining. Cells were then stained with mouse anti-E-Cadherin antibody (Abcam, USA) or anti-Collagen I antibody, and then stained with Alexa Fluor 488 goat anti-mouse IgG or 594-conjugated goat anti-rabbit IgG (Invitrogen, USA) with DAPI counterstaining. Samples were examined by a fluorescence microscope (Axio Observer, Zeiss, Germany).

### EV Labeling with NIR dye

IMRCs -EV were labeled with a near-infrared fluorescent dye. According to the manufacturer’s instructions for the ExoGlow™-Vivo EV Labeling Kit (Near IR) (EXOGV900A-1, System Biosciences, LLC., USA), 500 μl EV suspension containing approximately 250 μg protein equivalent (determined by BCA test) was incubated with NIR dye at room temperature for 45 min. Labeled EVs were recovered with 167 μl ExoQuick-TC. After incubation, labeled EVs were centrifuged at 13,000 g for 10 min to remove excess dye and resuspended in PBS buffer for in vivo imaging.

### In vivo imaging of the biodistribution of IMRCs-EVs delivered by different routes

To analyze the distribution of IMRC-EVs, NIR-labeled IMRC-EVs were delivered via different routes at 24 h after BLM exposure, and the mice were anesthetized and imaged by a small animal live imaging system MIIS (Molecular Devices, LLC., CA, USA) at 3, 9, 12, 24, 48, 96, 120 and 144 h post-treatment. In order to reduce autofluorescence, the ideal filter condition for NIR imaging was set at the near-infrared range (excitation 710 nm, emission 760 nm), and MetaMorph-MIIS software was used to analyze the data. The organs harvested at 24 h after injection of EVs via tail vein were imaged on the Odyssey CLx imaging system using 700 nm channel and analyzed using ImageStudio software (LI-COR Biosciences).

### Detection of cytokines in lungs

Lung tissue extracts were prepared as described previously [12]. The concentrations of cytokines such as TGF-β, TNF-α, IL-10, IL-6, IL-13, MCP-1, MIP-1α and VEGF in lung lysates were quantified by ELISA assay (ExCell Bio, China) according to the manufacturer’s instructions.

### Western blotting

Total protein samples were extracted from mouse lung tissue or cells with RIPA lysis buffer (P0013b, Beyotime, China) containing protease inhibitors (HY-K0010, MCE, USA). BCA Protein Assay Kit was used for total protein quantification. 30 μg proteins were separated by gel electrophoresis on a 4–15% precast gel (P0465S, Beyotime, China) and then transferred to PVDF membrane (IPVH00010, Millipore, USA). The membranes were blocked at room temperature with 5% milk for 1 h and incubated overnight at 4 °C with primary antibodies, then incubated with secondary antibodies at room temperature for 1 h. The ECL reagent was used to develop protein bands, and images were acquired using the BIO-RAD imager. The ImageJ system was used for quantification. The antibodies used for Western blotting were α-SMA, type I collagen, E-cadherin (E-cad), N-cadherin (N-cad) and fibronectin (FN) (Abcam, USA), Smad2/3 and p- Smad2/3 (CST, USA).

### Pulmonary function test (PFT)

Twenty-one days after BLM or saline treatment, mice were anesthetized with 50 mg/kg pentobarbital i.p. (Sigma, USA), and tracheostomy was performed. Animals were intubated with a 14-gauge cannula and connected to a Flexivent rodent ventilator (SCIREQ Inc., Canada).

### Computed tomography

Micro-CT imaging was performed in anesthetized mice (2–5% isoflurane/O2 gas) utilizing the Quantum GX Micro CT (PerkinElmer, Inc., MA). The heart-respiratory gating strategy was carried out. Voltage was set at 90 kV and the current was set at 200 μA and the images were captured over a 4 min interval. 2D images were obtained for visualization and display. The structural parameters for lung were analyzed using InForm software (PerkinElmer, Cambridge, UK).

### Real-time PCR

TRIzol reagent (Invitrogen, USA) was used to extract total mRNA from lung tissue and cells. mRNA was reverse transcribed using a Strand cDNA Synthesis SuperMix for qPCR kit (11141ES60, Yeasen, China). qPCR SYBR Green Master Mix kit (11196ES50, Yeasen, China) was used to amplify the cDNA in the Bio-Rad IQ5 system (Bio-Rad, USA). GAPDH was used for internal standardization. Primers for real-time PCR are shown in Additional file 5: Table S1.

### RNA-seq library preparation and sequencing

Three biological replicates were used for RNA-seq analyses. The TruSeqTM Stranded Total RNA Kit (Illumina, San Diego, USA) and SuperScript double-stranded cDNA synthesis kit (Invitrogen, USA) were used to remove ribosomal RNA (rRNA) and synthesize cDNA. Illumina HiSeq X Ten sequencing platform was used to perform high-throughput sequencing. The sequencing read length was paired-end (PE) 150, and a 15G rRNA library was constructed.

### Transcriptomic bioinformatic analysis

The FastQC software (v0.11.5) was used to evaluate the quality of the original sequencing data of each sample. Trimmomatic software (v0.36) was used to remove the adapter sequence and low-quality bases to obtain clean reads. The HISAT2 software (v 2.2.1.0) was used to align the reads to the reference genome and obtain the mRNA expression matrix. Principal component analysis (PCA) of the BLM-induced pulmonary fibrosis and gene expression levels of two EV treatment groups from different routes was performed. The R package Stats (v4.0.3) and Ggord (v 1.1.5) were used to perform PCA on the regularized log2 transformed data. The first two principal components were drawn. The R package DESeq2 (v1.28.1) was used to normalize the original quantitative data and analyze differential genes, and genes whose multiples of change were greater than 2 times were retained. *P*-value < 0.05 was considered statistically significant. Principal component regression analysis was used to obtain the transcriptome overlap slopes of the two treatment groups relative to the disease model group, and the slopes were used to evaluate the relative treatment effect quantitatively. Before the regression analysis, all input values were used to calculate the base 2 logarithmic values (log2FoldChange, log2FC) of the gene expression changes of the BLM group and the two treatment groups compared with control group, and scatter plots were drawn. Taking the transcriptomic gene expression changes in the BLM group as the base (X-axis), the principal component regression was used to calculate the transcriptome overlap slopes of the groups of different treatment routes relative to the BLM group, and the regression lines and slopes were displayed in the scatter plot. The R package EnrichR (v3.0) was used to perform GO enrichment analysis of the differentially expressed genes. Gene expression heat maps were drawn on the website http://www.ehbio.com/Cloud_Platform/front/#/.

### Statistical analysis

All results are expressed as the mean ± standard deviation. A one-way analysis of variance (ANOVA) after Shapiro–Wilk normality test for normality and Bartlett's test for the homogeneity of variance was used for comparison between groups. P < 0.05 was considered statistically significant. Statistical analysis was performed using GraphPad Prism 8.0 software (GraphPad Software, Inc.).

## Results

### Separation and identification of EV

**Fig. 1:**
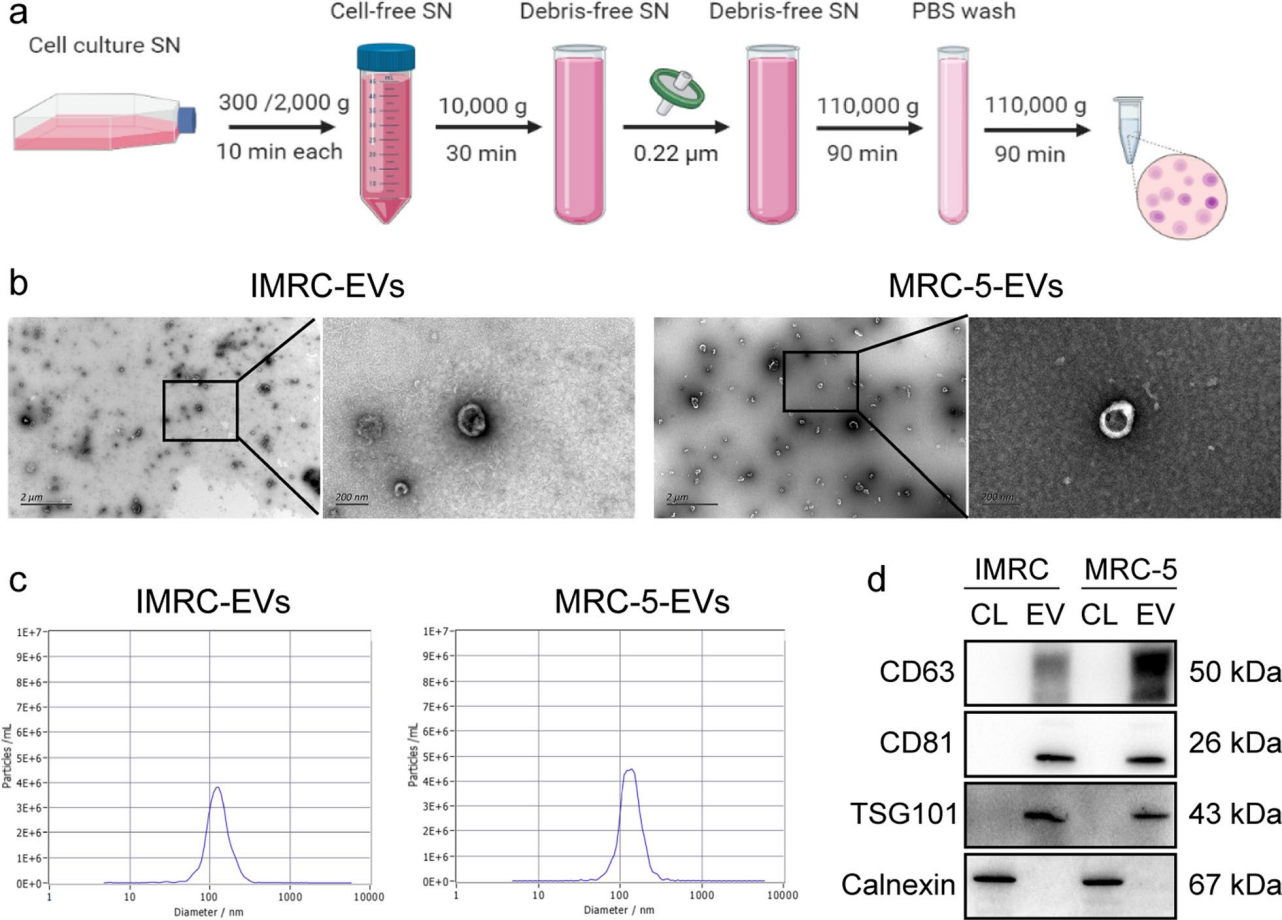
Characterization of EVs. **a** Process of differential ultracentrifugation to purify EVs. **b** Representative transmission electron microscopy image of EVs. Left scale bar = 2 μm. Right scale bar = 200 nm. **c** Nanoparticle tracking analysis showed the size and distribution of EVs purified by differential ultracentrifugation. The average size of EVs is 120 nm. **d** Representative Western blot analysis of the exosomal marker CD63, CD81 and TSG101, and the endoplasmic reticulum marker calnexin in EVs derived from IMRCs and MRC-5 cells. Cellular lysate (CL) was used as a control

We collected the culture supernatants of IMRC and MRC-5 to extract EVs by differential ultracentrifugation (Fig. 1a). The morphology of EVs was observed through transmission electron microscopy. EVs from IMRC or MRC-5 had a classic clear membranous saucer-like structure (half-recessed on one side), with a complete envelope and low-density particulate matter inside (Fig. 1b). NTA was performed to analyze the number and purity of EVs. The results showed that the vesicle size distribution had a single peak. The particles with a diameter of approximately 120 nm in the centrifugal sediment accounted for the vast majority of the particles in the sample (Fig. 1c and Additional file 6: Table S2). We did not find EVs larger than 1000 nm, indicating that there were no apoptotic bodies in our samples, and the cells were in good condition. We performed EV marker protein verification according to the MISEV2018 guidelines [18]. Tetraspanins CD63, CD81, and TSG101 were positive in the EVs; and the endoplasmic reticulum marker calnexin was negative in the EVs (Fig. 1d). These results suggested that EV proteins met the requirements of “three positives and one negative results” proposed in the guidelines, and the sample purity was high.

### IMRC-EVs can prevent BLM-induced pulmonary fibrosis in mice

**Fig. 2:**
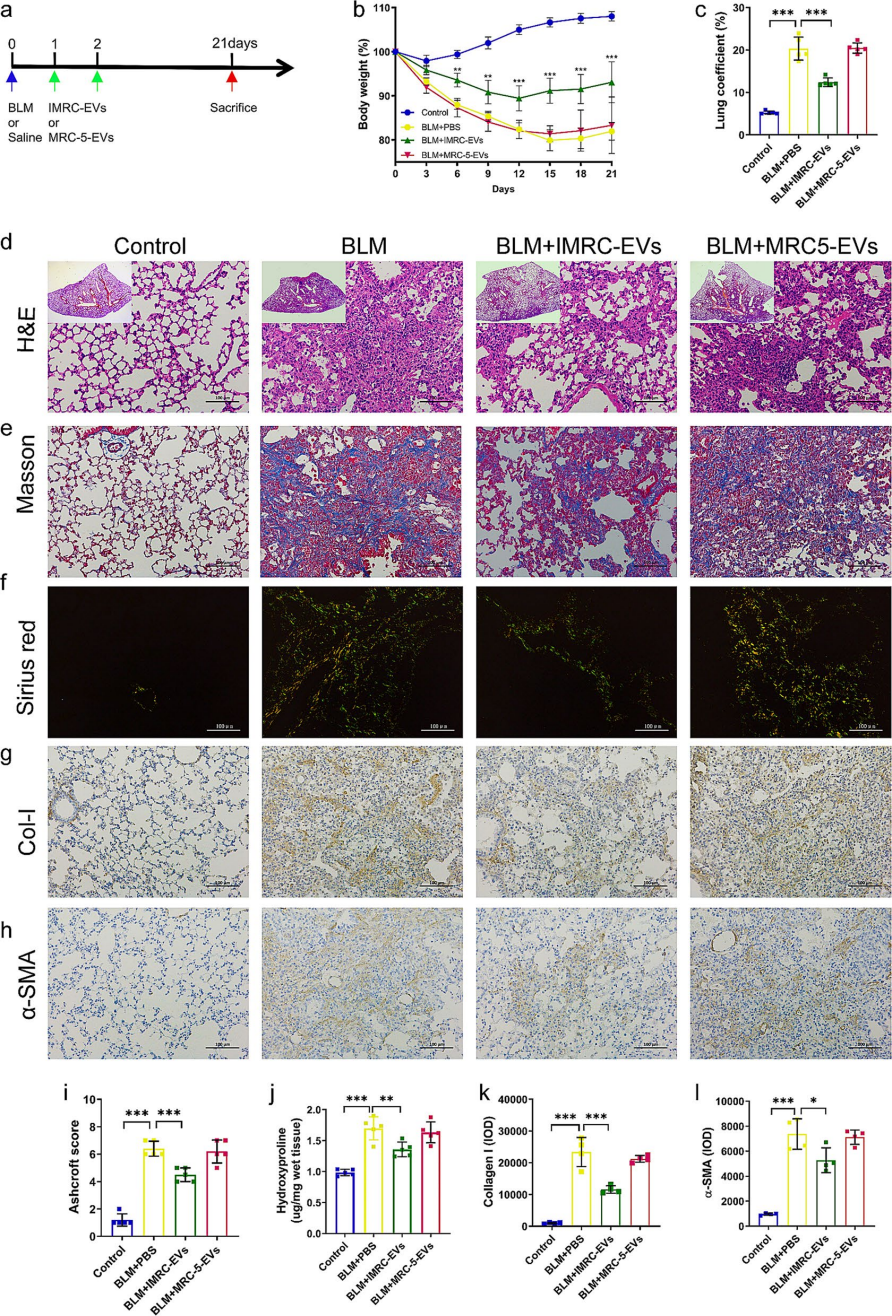
Therapeutic effect of IMRC-EVs on BLM-induced lung fibrosis mouse model. **a** Schematic diagram of the in vivo experimental design of the BLM-induced PF mouse model. **b** Changes in relative body weight (%) of mice receiving different interventions. **c** Lung coefficient (wet lung weight/total body weight) of all treatment groups. **d–f** Mouse lung tissues from each group were collected and embedded in paraffin for histological analysis. **d** Representative histology of lung sections stained with H&E. The small graphs inside the figure represent whole lung from all groups at day 21 post-injury. **e** Representative Masson staining. myofibers (red), collagen fibers (blue), and nucleus (black-purple). **f** Representative picrosirius red staining. Scale bar = 100 μm. Collagen I displays an orange-red birefringence under polarized light, whereas Collagen III has a green birefringence. **g, h** Immunohistochemical (IHC) analysis of Collagen I (col-I) (**g**), and α-smooth muscle actin (α-SMA) (**h**) after IMRC-EVs and MRC5-EVs transplantation. Original magnification is ×200, scale = 100 μm. **i** Quantification of fibrosis by Ashcroft score. Ashcroft score was measured by averaging the score from a blinded and a non-blinded scorer. **j** Changes in hydroxyproline levels of the lung in different treatment groups. **k, l** The quantification of relative immunostaining of Col-I (**k**) and α-SMA (**l**). Data are represented as the mean ± SD; *P < 0.05, **P < 0.01, ***P < 0.001

We first injected different dosages of IMRC-EVs into the mice to evaluate their liver and kidney toxicity. The data showed that there were also no significant changes in the levels of AST, ALT, blood urea nitrogen and CREA, which indicated that the IMRC-EVs had no toxic effect on the liver and kidney function in mice (Additional file 7: Table S3). A mouse model of BLM-induced pulmonary fibrosis was used to evaluate the functional effects of IMRC-EVs on lung injury and fibrosis (Fig. 2a). Mice injected with BLM lost weight sharply in the first two weeks onward compared to control mice. Transplanting with IMRC-EVs, but not MRC5-EVs, ameliorate the loss of weight induced by BLM throughout the course of the pathology (Fig. 2b), improved lung coefficient (Fig. 2c), protected lung architectures (Fig. 2d, e), decreased fibrosis (Fig. 2i) and collagen deposition (Fig. 2f, j). Parallelly, immunohistochemical staining showed increased expressions of Col-I and α-SMA in BLM-treated mice, and intravenous administration of IMRC-EVs but not MRC5-EVs after BLM exposure reduced the expression of Col-I and α-SMA in the lungs (Fig. 1g, h, k, l).

### In vivo distribution of IMRC-EVs delivered by different routes

**Fig. 3:**
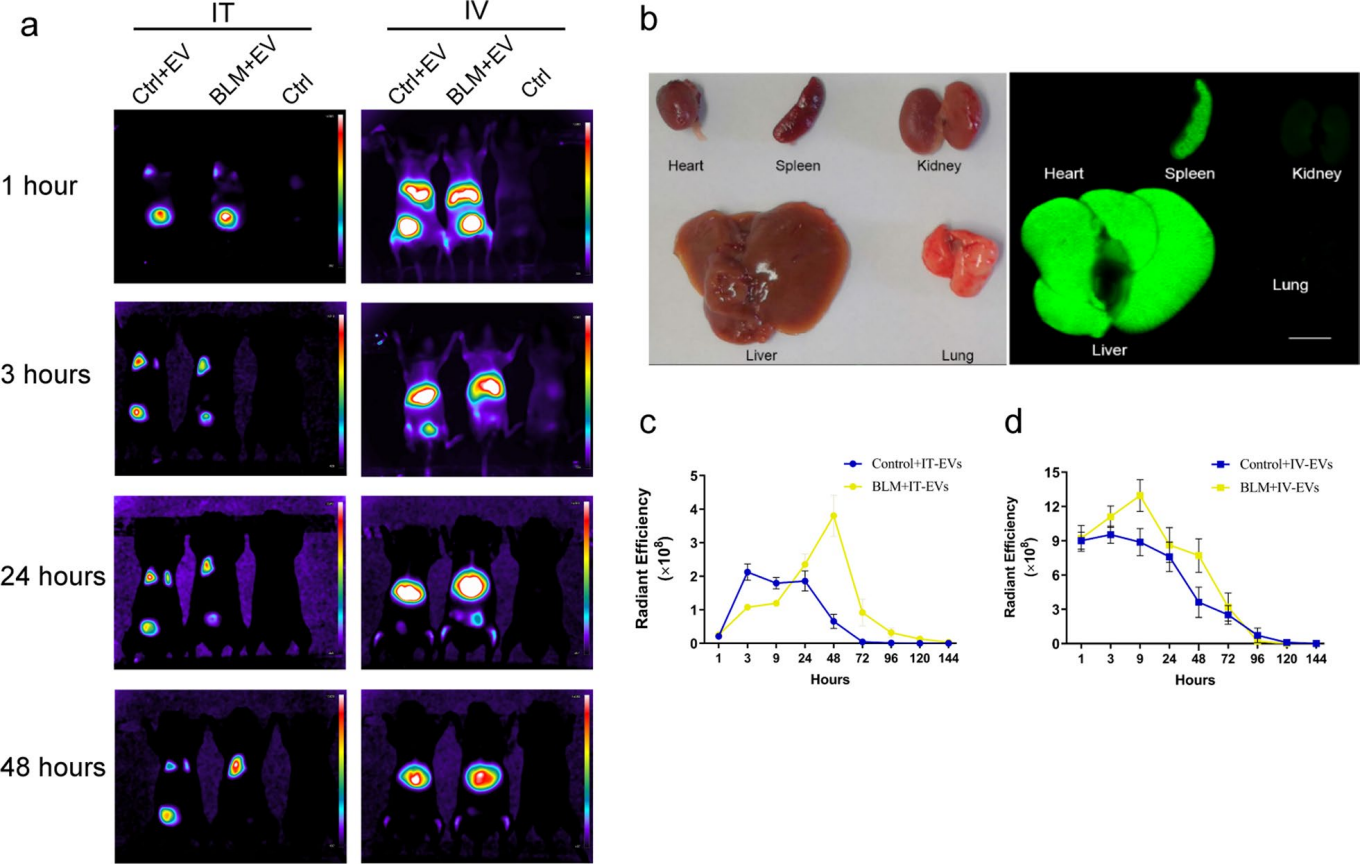
The distribution of IMRC-EVs delivered by different routes. **a** Live animal imaging of NIR fluorescence after IMRC-EV injection via different routes. IMRC-derived EVs were labeled with NIR and injected into normal mice and BLM- induced mice via the IT or IV route. Mice in the control group were injected with equal volumes of normal saline. **b** Representative images of organs (24 h post-injection) from BLM-induced mice injected i.v. with IMRC-EVs. **c, d** The change trend of the fluorescence intensity signal at different time points after the injection of NIR-labeled IMRC-EVs via the IT route (**c**) and IV route (**d**)

To assess whether the injection route affects the distribution pattern of EVs, mice were given NIR-labeled IMRC-EVs via the IT or IV routes. In vivo fluorescence images show that in the tracheal infusion group, the strong fluorescence was mainly distributed in the lung region, whereas in the tail vein infusion group, it was mainly distributed in the liver and spleen, suggesting that IMRC-EVs retained in different organs after injection via different routes (Fig. 3a, b). For both delivery routes, the fluorescent signal intensity was higher, and the retention time was longer in BLM-treated mice compared to control mice (Fig. 3c, d). In the IT route, the fluorescence signal in the BLM group took longer than in the control group to reach the peak. The reason may be that the permeation and retention effect of the injured lung made EVs more difficult to diffuse into the lungs and give fluorescent signals. These results indicated that different delivery routes generated different biodistribution patterns. Thus, targeted tissue distribution may be achieved by specific delivery route.

### Both routes of IMRC-EVs delivery can regulate the early cytokine levels of BLM-induced pulmonary fibrosis in mice

**Fig. 4:**
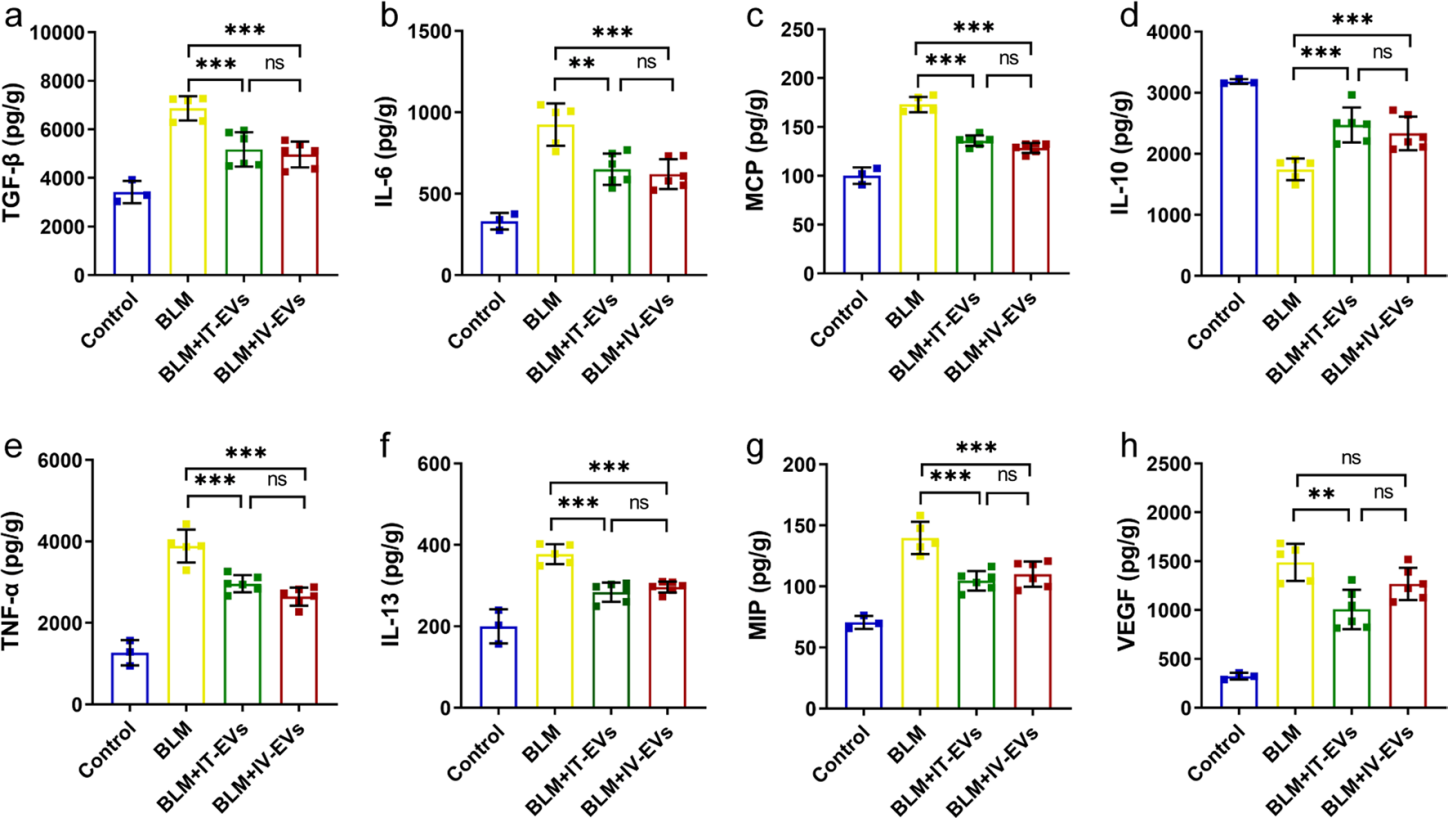
The effect of IMRC-EVs delivered via the tracheal and intravenous routes on cytokine levels in the acute phase of the BLM-treated mice. Five days after BLM injury, mice were euthanized and cytokine levels in lung lysate were analyzed by ELISA (**a–h**). Data are represented as the mean ± SD; n = 5. *P < 0.05, **P < 0.01, *** P < 0.001, and ns indicates no difference. TGF-β1 transforming growth factor-β1, IL interleukin, MCP Monocyte chemoattractant protein, TNF-α tumor necrosis factor-α, MIP macrophage inflammatory protein and VEGF vascular endothelial growth factor

Since preceding inflammation is important for the formation of BLM-induced pulmonary fibrosis [21], we examined whether IMRC-EVs could modulate the inflammatory cytokine level in the lungs via different delivery routes. Compared with the BLM-treated group, IMRC-EVs delivered by both the IT and IV routes down-regulated the expression of pro-inflammatory factors (e.g., TGF-β1, TNF-α, IL-6, IL-13), chemokines (e.g., MCP, MIP), VEGF and slightly increased the levels of the anti-inflammatory cytokine IL-10 (Fig. 4). These results indicated that different delivery routes of IMRC-EVs exert anti-inflammatory and immunomodulatory activities, which may be one of the mechanisms that ameliorate BLM-induced acute lung injury and delay the progression of fibrosis.

### Therapeutic effects of IMRC-EVs delivered by different routes in mouse models of pulmonary fibrosis

**Fig. 5:**
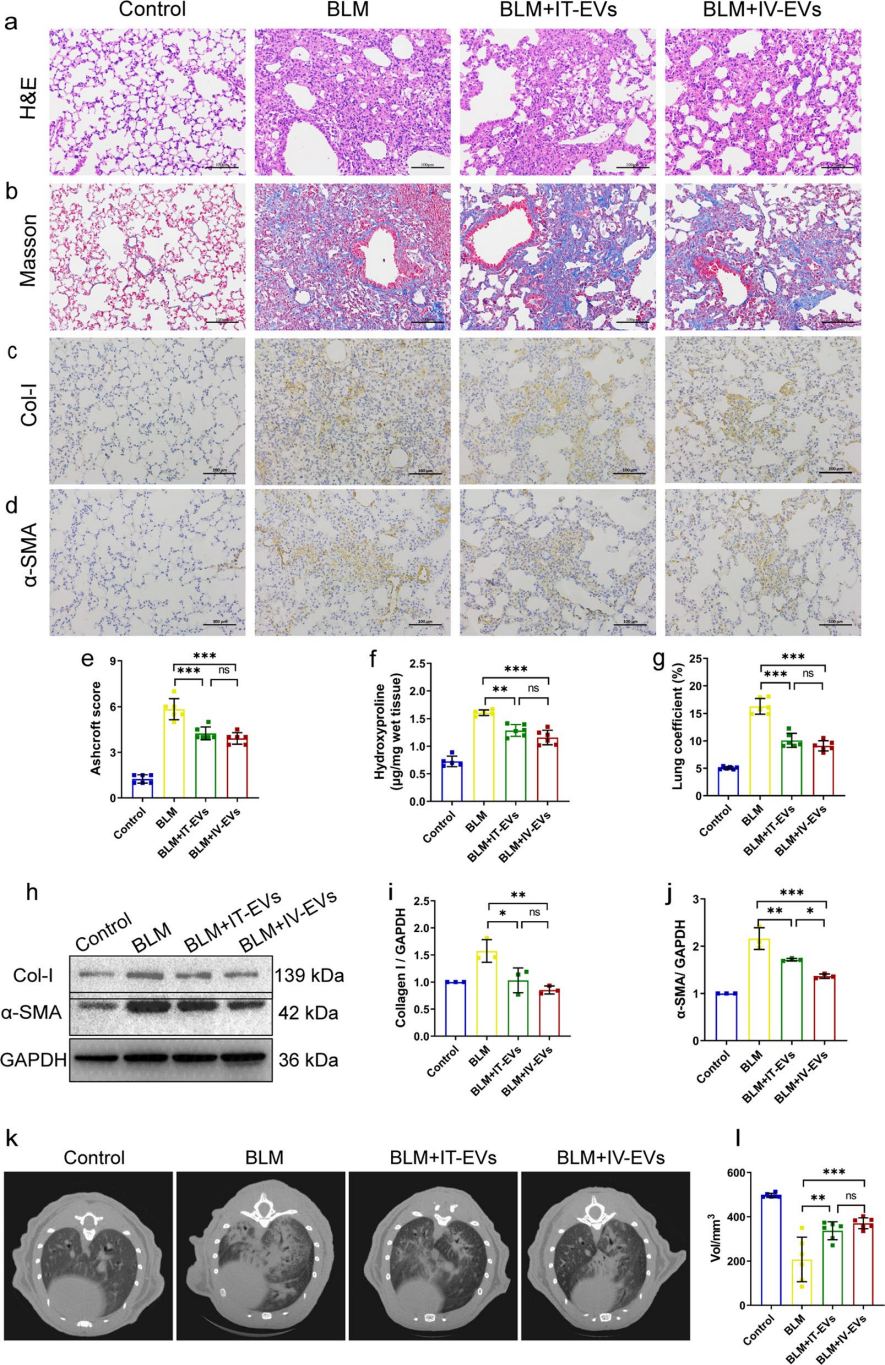
Inhibition of pulmonary fibrosis by IMRC-EVs delivered via different routes in a mouse model of BLM-induced pulmonary fibrosis. **a**, **b** H&E staining (**a**) and Masson staining (**b**) micrographs of paraffin sections of mouse lung tissue in each group. The blue filaments represent collagen fibers, indicating interstitial fibrosis. **c, d** Representative photomicrographs of Col-I (**c**) and α-SMA (**d**) immunostained sections from each group. **e** Semi-quantitative assessment was performed on day 21 using Ashcroft scoring method. **f** The content of hydroxyproline (HYP) was determined in lung tissues, which is a marker of collagen deposition. **g** The lung coefficients of mice in each group. **h** Western blot for Col-I and α-SMA protein expression in lung tissue from each group. Quantification of the relative Collagen I protein expression levels (**i**) and α-SMA protein expression levels (**j**). **k** Representative microCT image 21 days after BLM instillation. **l** Quantitative CT imaging was used to measure the lung volume (n = 4–6). The data are expressed as the mean ± SD.*P < 0.05, **P < 0.01, ***P < 0.001, and ns indicates no difference

We examined the effects of IMRC-EVs delivered by different routes on lung repair and fibrosis. Mice treated with BLM showed fibrosis formation, which was reduced after IMRC-EVs delivery via tracheal or intravenous routes. Both administration routes showed therapeutic effects in maintaining normal lung architecture (Fig. 5a), decreasing fibrosis (Fig. 5b, d, e, g) and collagen deposition (Fig. 5c, f). We then investigated the expression of col-Ι and α-SMA in the lung tissues affected by different routes of IMRC-EVs delivery (Fig. 5h–j). Notably, the expression of α-SMA in the intravenous group was significantly reduced compared with BLM-treated group, and lower than the tracheal group (Fig. 5j), indicating that the intravenous delivery route was superior to the tracheal delivery route in terms of inhibiting the activation of fibroblasts and α-SMA expression in the mouse lung tissue. Furthermore, MicroCT showed obvious fibrotic lesions in mice on the 21st day after BLM treatment. Both routes of IMRC-EVs delivery could improve this situation, and there was no statistical difference between the two groups. Both routes of IMRC-EVs delivery could similarly improve the lung functions, which were impaired by BLM treatment (Additional file 1: Figure S1).

### Potential mechanisms of IMRC-EVs delivered by different routes

**Fig. 6:**
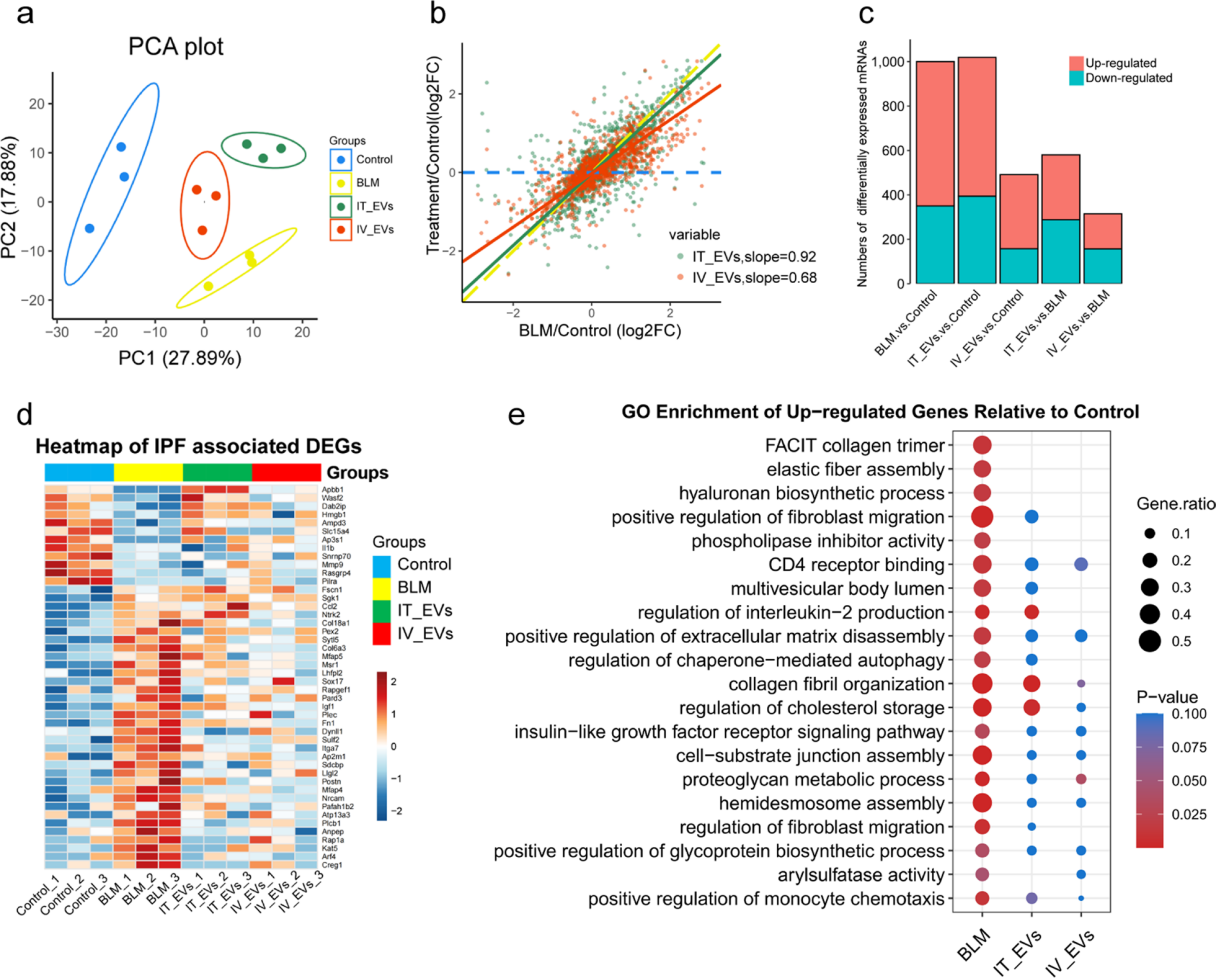
Analysis of transcriptome changes in BLM-induced pulmonary fibrosis and the EV treatment groups through IT or IV routes. **a** Principal coordinates analysis (PCA) of transcriptome differences among the Control, BLM, IT_EVs and IV_EVs groups. Colors and shapes indicate different groups. Ellipses indicate the 95% confidence interval. **b** Comparison of the slopes among BLM, control groups and different pathways of EVs delivery groups. **c** Differentially expressed mRNAs among the four groups. **d** Gene expression heatmap across different groups for the DEGs. Heatmap colors indicate normalized gene expression ranging from high (red) to low (blue). **e** The bubble chart of GO enrichment of pairwise differentially expressed genes. The color of bubbles indicates the P-value, and the radius of bubbles is the gene ratio. Gene ratio, the percentage of DEGs in the enriched gene set

We performed RNA sequencing of the lung tissues harvested on the 21st day post BLM treatment to explore the possible therapeutic mechanisms. All sequencing data underwent stringent quality control measures and normalization prior to analysis (Additional file 2: Figure S2). PCA showed that the samples in each group had sound clustering, and the groups could be separated clearly, suggesting that the samples within each group had sound reproducibility (Fig. 6a). Unlike PCA, the value of the overlap slope provides a relatively quantitative evaluation index for treatment, which helps to roughly understand the treatment effect and the degree of improvement. As shown in Fig. 6b, comparison of the slopes between different groups indicates a gradient of transcriptomic severity. The slopes of the log2-FC effect sizes for IT-EVs and IV-EVs were 0.92 and 0.68, respectively, indicating that the transcriptome deviation of IV-EVs was further than IT-EVs compared with BLM-treated group. Pairwise differential expression analysis showed that a large number of differentially expressed genes appeared after BLM induction. After EVs treatment, gene expression changed significantly (Fig. 6c). Heatmap also indicated that remarkable differences of mRNAs occurred between BLM and control groups as well as IT-EVs and IV-EVs-treated mice (Fig. 6d). Among these differentially expressed mRNAs, the expression of a panel of fibrotic genes were significantly altered after the treatment with IMRC-EVs through both routes, such as Fn1, Igf1, Col6a3, Mmp9 and Postn (Additional file 8: Table S4). Furthermore, we performed GO analysis to explore whether the differentially expressed mRNAs were enriched in certain biological functions. Enrichment analysis revealed that both EVs delivery routes regulate collagen trimer production, elastic fiber assembly, hyaluronan biosynthetic process and phospholipase inhibitor activity. However, the IV route has a stronger role in the regulation of fibroblast migration, multivesicular body lumen, regulation of interleukin-2 production, regulation of chaperone-mediated autophagy, etc., while the IT pathway has a stronger role in the arylsulfatase activity (Fig. 6e, Additional file 3: Figure S3). According to the above results, we suggest that the IT and IV pathways differ in their mechanisms of reducing pulmonary fibrosis.

### IMRC-EVs inhibit EMT of A549 cells induced by TGF-β1

**Fig. 7:**
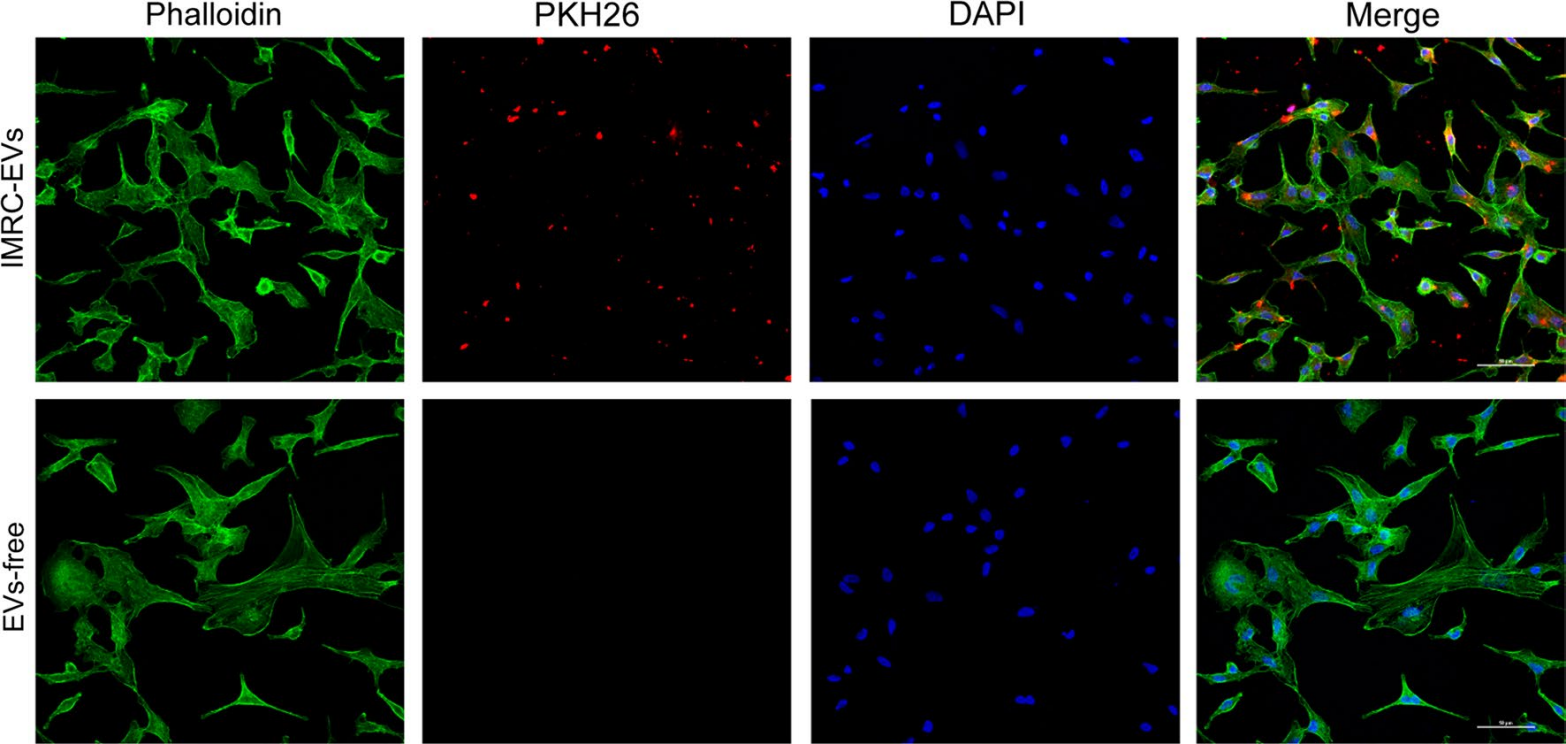
A549 cells were co-cultured with IMRC-EVs. A549 cells were incubated with PKH26 fluorescently labeled IMRC-EVs for 4 h. A549 cells were stained for actin (phalloidin, green) and nuclei (DAPI, blue). Scale bar, 50 μm

Since alveolar epithelial cell injury is considered to be the initiation event of pulmonary fibrosis, and EVs delivered via tracheal route can retain in the pulmonary tissues, we asked whether EVs can be internalized by alveolar epithelial cells that may contribute to pulmonary fibrosis through EMT. We labeled EVs with the membrane dye PKH26 and co-incubated them with a pulmonary epithelial cell line A549 [22]. Confocal microscopy analysis showed that the labeled EVs appeared in the cytoplasmic and perinuclear regions of the epithelial cells (Fig. 7), indicating that EVs could be internalized by alveolar epithelial cells. We also noticed that the size of PHK26 signals were heterogeneous, suggesting that EVs ingested by the cells may be presented in single or different size of aggregates. Consistent with previous reports, MSC-EVs can be effectively internalized by lung epithelial cells and exert a protective effect [23, 24].

**Fig. 8:**
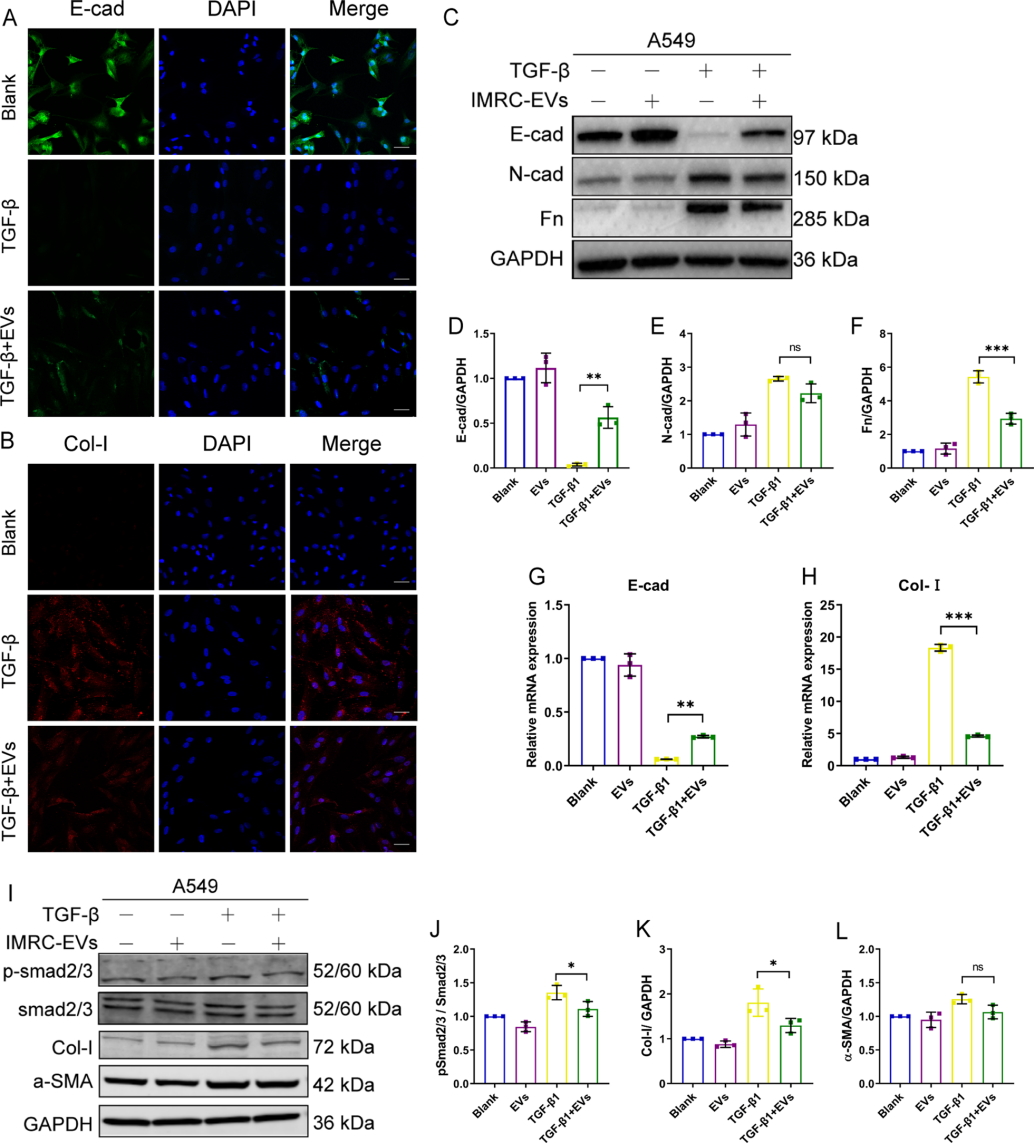
IMRC-EVs inhibit TGF-β-induced EMT in vitro. Immunofluorescence staining of E-cad (**a**) and Col-I (**b**) expression in A549 cells with or without the treatment with 2 ng/mL TGF-β and IMRC-EVs for 48 h. Scale bar, 20 μm. **c** Western blot for E-cad, N-Cad and Fn protein expression in A549 cells, with or without 2 ng/mL TGF-β1 and IMRC-EVs treatment for 48 h. GAPDH was used as a loading control. **d**–**f** Quantification of the relative protein expression levels in lung tissues from mice relative to GAPDH. **g**, **h** qPCR for the relative expression of E-cad and Col-I mRNA in A549 cells, with or without 2 ng/mL TGF-β1 and IMRC-EVs treatment for 48 h. **I** The expressions of Smad2/3, active p- Smad2/3 factors, α-SMA and collagen-I were determined by Western blotting. The relative phosphorylation ratio is determined by p-Smad2/3 vs. total Smad2/3 and was presented graphically (**j**). Quantification of the relative Collagen I protein expression levels (**k)** and α-SMA protein expression levels (**l**)

We next studied the functional consequences of IMRC-EVs uptake by epithelial cells in vitro. A549 cells were cultured in the presence or absence of IMRC-EVs, along with TGF-β1 treatment to induce EMT. Our results showed that IMRC-EVs inhibited the morphological changes of A549 cells induced by TGF-β1 (Additional file 4: Figure S4). Immunofluorescence and PCR analysis further confirmed that IMRC-EVs inhibited col-I expression during the transdifferentiation of A549 and preserved some degree of E-cad expression (Fig. 8a, b, g, h). There were similar results for N-cad, Fn and E-cad as revealed by Western blotting (Fig. 8c-f). These findings suggest that EV has a protective effect on lung epithelial cells by inhibiting the EMT process of damaged epithelial cells.

Since Smad2/3 is a key downstream mediator of TGF-β-induced anti-proliferative effect in many cell types [25], levels of activated/phosphorylated forms of Smad2/3 (p-Smad2/3) and total Smad2/3 were assessed by Western blot. The results demonstrated that TGF-β1 could increase the ratio of p-Smad2/3/Smad2/3, indicating of Smad2/3 activation. Our results showed that the IMRC-EVs suppressed the TGFβ1-induced Smad2/3 phosphorylation and ameliorated the induction of Collagen I, α-SMA expression (Fig. 8i–l). These results suggested that IMRC-EVs might exert anti-fibrotic activity via repressing the TGFβ/Smad pathway.

## Discussion

Organ fibrosis is the main cause of disability and death from many diseases worldwide. Recently, researchers have found that MSCs exhibit good anti-fibrosis effects in a variety of experimental fibrosis models. Numerous countries around the world have carried out phase I clinical trials on MSCs for the treatment of IPF to evaluate the safety of stem cell therapy [26, 27]. EVs derived from MSCs are considered as a potential method for the treatment of fibrotic diseases[15, 28]. Therefore, we extracted EVs from IMRCs to further evaluate their tissue repair and anti-inflammatory, anti-fibrosis and immune regulation effects.

Bleomycin is currently the most commonly used drug to induce pulmonary fibrosis[29]. In the present study, we constructed the guideline-recommended mouse model of bleomycin-induced pulmonary fibrosis. Nevertheless, there are some limitations in this fibrotic model that should be noted, such as the time course of disease development being different from that monitored in humans. In humans, fibrosis is the result of repeated damage and abnormal repair of the alveolar epithelium eventually causing progressive irreversible fibrosis. Bleomycin-induced lung injury can be divided into two phases, the acute lung injury phase and the subsequent lung fibrosis phase. Moreover, lung injury in mice is self-limiting, and fibrotic lesions could regress. Therefore, although in the present study we attempted to investigate the effect of IMRC-EVs on early pulmonary fibrosis and also preliminarily evaluated the effect of IMRC-EVs on the TGF-β activation pathway at the in vitro level, whether EVs can have any beneficial effect in patients with pulmonary fibrosis still needs to be carefully investigated, and the effective EVs administration time window and the related molecular markers need to be further explored.

Our research shows that early intervention of IMRC-EVs can improve pulmonary function, decrease hydroxyproline levels, inhibit the degree of pulmonary fibrosis, and slow the development of pulmonary fibrosis in BLM-induced mice. In a model of silicosis, both adipose-tissue-derived MSCs and EVs reduced collagen fiber content and the expression of inflammatory mediators in lung tissue, with a higher dose of EVs achieving a therapeutic effect comparable to that of MSCs [30]. Furthermore, Willis et al. found that MSC-EVs treatment was effective in improving hyperoxia-induced pulmonary fibrosis and restoring lung function in mice. The above evidences support that stem cell-derived EVs have anti-fibrotic effects and that they can be used as a cell-free intervention alone for the treatment of IPF [15]. Interestingly, we found that IMRC-EVs were efficacious, whereas EVs derived from fibroblasts did not show therapeutic effects. Similarly, Tan et al. [21]. compared human amniotic epithelial cell-derived exosomes (hAEC Exo) with human lung fibroblast exosomes. Intranasal administration of hAEC Exo, but not fibroblast exosomes, on the first day after bleomycin challenge reduced lung inflammation, while treatment on the seventh day reduced fibrosis. These results indicate that EVs may possess similar functions as parental cells.

Inflammation is a defense response of the body to stimuli, an important process in the development of tissue damage. When this process continues, the development of a chronic inflammatory state will promote fibrosis formation by increasing proliferation of fibroblast [31, 32]. Recent studies have shown that pulmonary fibrosis can be alleviated by altering the inflammatory Milieu [33]. MSCs have potent anti-inflammatory, immunomodulatory properties and reduce tissue damage through paracrine release of multiple types of cytokines [34, 35]. A common observation is that MSC treatment reduced the expression of pro-inflammatory cytokines (including IL-1α, IL-1β, IL-6, IFN-γ and TNF- α), pro-fibrotic factors (TGF-β) and chemokines (MIP, MCP) and increased the expression of anti-inflammatory cytokines (e.g. IL-10) in both the peripheral blood and local pulmonary tissues [36, 37]. Our study showed that IMRC-EVs regulated the levels of multiple cytokines in the tissues of BLM-induced lung injury in the acute inflammatory period after BLM instillation. This confirms that IMRC-EVs have the same anti-inflammatory and immunomodulatory effects as the parent cells and that they have a protective effect on BLM-induced acute lung injury [12]. Notably, in the MSC-EVs-treated bronchopulmonary dysplasia (BPD) model, the investigators observed an upregulation of VEGF expression [24]. However, we found that IMRC-EVs treatment suppressed the expression level of VEFG in lung tissue during the acute phase, which is similar to previous findings [38]. The mechanism of MSC-EVs in different lung disease models deserves further investigation.

Some studies have suggested that the tissue distribution of EVs may be influenced by the route of administration [39]. It has been reported that adipose-derived MSC-EVs injected by the tracheal route can first reach the alveoli, and were mainly concentrated near the airway in a silica-induced silicosis model. Both healthy areas and intensely inflammatory regions presented fluorescence [30]. However, the biodistribution of MSC-EVs in a BLM-induced lung fibrosis model has not been previously reported. Therefore, we performed the in vivo biodistribution experiments to determine whether EVs reached the target tissues or whether they had pleiotropic effects by affecting multiple tissues and organs. In this study, tracing EVs labeled with lipophilic dyes via in vivo optical imaging, we demonstrated that transtracheal delivered IMRC-EVs successfully reached the lung parenchyma. In addition, we also investigated the distribution of EVs in vivo after IV instillation, with the highest accumulation of EVs detected in the liver and spleen. No fluorescent signal was detected in the lungs, which may be due to the detection threshold of the technique. As demonstrated by several studies, intravenously injected EVs are initially mainly located in the liver and spleen, and then, are rapidly redistributed to the gastrointestinal tract and lungs; they are then cleared by the kidneys and liver [39, 40]. These results together demonstrated that the liver and spleen were the dominant resident organs after EV injection through the vein.

There is no consensus yet on the optimal route of administration of MSC-EVs. For this purpose, we evaluated the different administration routes (IT and IV) of EVs in adult mice. The most widely used strategy for EV in vivo delivery is the IV route, which is considered the fastest way to deliver EV due to their direct delivery to the systemic circulation [41]. Like many treatments, repeated administration is necessary for chronic diseases. The IV route is preferred possibly due to the simple and practical procedures that allow repeated transplantation. In contrast, the advantage of intratracheal instillation for local administration is that it can enter the target tissue immediately, improve the utilization rate, and reduce the side effects to other organs (off-target effects). In lung diseases, the delivery of MSCs via the IT route has shown a therapeutic effect in the IPF model [42, 43]. In our study, we found that both administration routes of IMRC-EVs were effective in preventing fibrosis when compared with the saline control group, and no significant difference was found in their therapeutic effects, indicating that both local and systemic anti-inflammatory may be effective in the treatment of IPF, and the specific differences in mechanisms need to be further investigated.

As aforementioned, the different EVs delivery routes determine the different organs in which MSC-EVs reside, and the different cell types they first encountered when entering the body, which implies different mechanisms for their therapeutic effects. Our transcriptome results showed that: both EVs delivery routes regulate collagen trimer production, elastic fiber assembly, hyaluronan biosynthetic process and phospholipase inhibitor activity. However, the IV route has a stronger role in the regulation of fibroblast migration, multivesicular body lumen, regulation of interleukin-2 production, regulation of chaperone-mediated autophagy, etc., while the IT pathway has a stronger role in the arylsulfatase activity. Accordingly, our data showed that IMRC-EVs could prevent EMT-induced phenotypic changes in epithelial cells, at least in vitro, and that IMRC-EVs also could reduce collagen levels during fibrogenesis induced by TGF-β1. After delivery of IMRC-EVs via IT route, IMRC-EVs may crosstalk with alveolar epithelial cells in the local microenvironment, thereby protecting epithelial cells from damage. This may be one of the mechanisms by which IMRC-EVs delivered via the tracheal route attenuates pulmonary fibrosis. Obviously, different delivery routes can affect the way MSC-EVs activate and initiate their anti-inflammatory procedure [44].

## Conclusion

IMRC-EVs have a powerful immunomodulatory effect and can protect normal alveolar structure, reduce collagen accumulation and reduce BLM-induced fibrosis. IMRC-EVs delivered by different routes generate a different biodistribution and may play a therapeutic role through different pathways. Importantly, our results proved that EVs derived from IMRCs can reproduce many of the protective effects of IMRCs and are candidates for cell-free therapy. Considering the safety and practicality, the clinical application of IMRC-EVs may be more favored than its parent cells for IPF in the near future.

## Supplementary Information

**Additional file 1: Figure S1.** Pulmonary function analysis after different routes of IMRC-EVs delivery.

**Additional file 2: Figure S2.** Evaluation of the RNA-seq data quality (a and b) Boxplot and expression quantity distribution density diagram among the four groups. (c and d) PCA and cluster analysis show that BLM-treated mice had different RNA expression profiles compared to control groups).

**Additional file 3: Figure S3.** GO enrichment of down-regulated genes relative to control group.

**Additional file 4: Figure S4.** IMRC-EVs reduce the pro-fibrotic effects of TGF-β1. Representative morphology of A549 cells, with or without 2 ng/mL TGF-β1 and IMRC-EVs treatment for 48 hours.

**Additional file 5: Table S1.** Primers used for PCR amplification.

**Additional file 6: Table S2.** Peak Analysis of IMRC-EVs and MRC5-EVs.

**Additional file 7: Table S3.** Liver and kidney toxicity of IMRC-EVs in mice.

**Additional file 8: Table S4.** List of differential Gene Expression in mouse lung tissue.

## Data Availability

The datasets generated and analysed during the current study are available in the NCBI SRA repository under this accession ID: PRJNA752772.

## Abbreviations

IPF: Idiopathic pulmonary fibrosis
IMRCs: Immunity-and-matrix-regulatory cells
IMRC-EVs: IMRC-derived extracellular vesicles
IT: Intratracheal
IV: Intravenous
MSCs: Mesenchymal stem cells
hESCs: Human embryonic stem cells
EV: Extracellular vesicle
BLM: Bleomycin
EMT: Epithelial-to-mesenchymal transition
TCM: Conditioned medium
DMEM: Dulbecco’s modified Eagle’s medium
TEM: Transmission electron microscope
NTA: Nanoparticle tracking analysis
BCA: Bicinchoninic acid
HYP: Hydroxyproline
Col-I: Collagen I
α-SMA: Alpha-smooth muscle actin
DAPI: 4′,6-Diamidino-2-phenylindole
TGF-β: Transforming growth factor β
TNF-α: Tumor necrosis factor α
IL: Interleukin
MCP-1: Macrophage chemoattractant protein-1
MIP-1α: Macrophage inflammatory protein 1α
VEGF: Vascular endothelial growth factor
E-cad: E-cadherin
N-cad: N-cadherin
FN: Fibronectin
PFT: Pulmonary function test
PCA: Principal component analysis
PE: Paired-end
ECM: Extracellular matrix
hAEC Exo: Human amniotic epithelial cell-derived exosomes
