# Supplementary figures and images for: Every road leads to Rome: therapeutic effect and mechanism of the extracellular vesicles of human embryonic stem cell-derived immune and matrix regulatory cells administered to mouse models of pulmonary fibrosis through different routes
Source: Stem Cell Res Ther. 2022 Apr 12;13:163. doi: 10.1186/s13287-022-02839-7 (PMC9006546; doi:10.1186/s13287-022-02839-7)

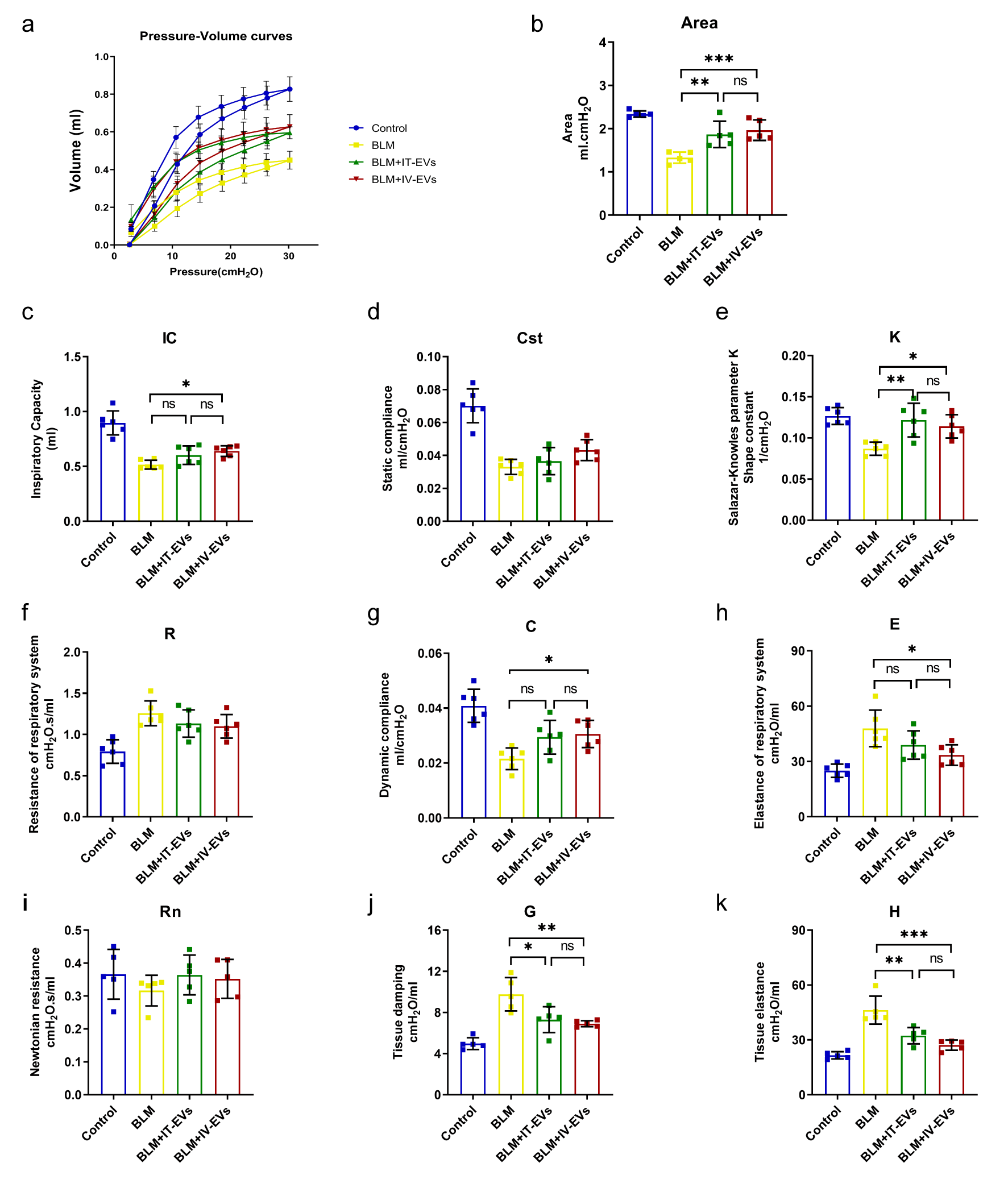

Supplement: Supplementary file 1 — Additional file 1: Figure S1. Pulmonary function analysis after different routes of IMRC-EVs delivery. [file 13287_2022_2839_MOESM1_ESM.tif]

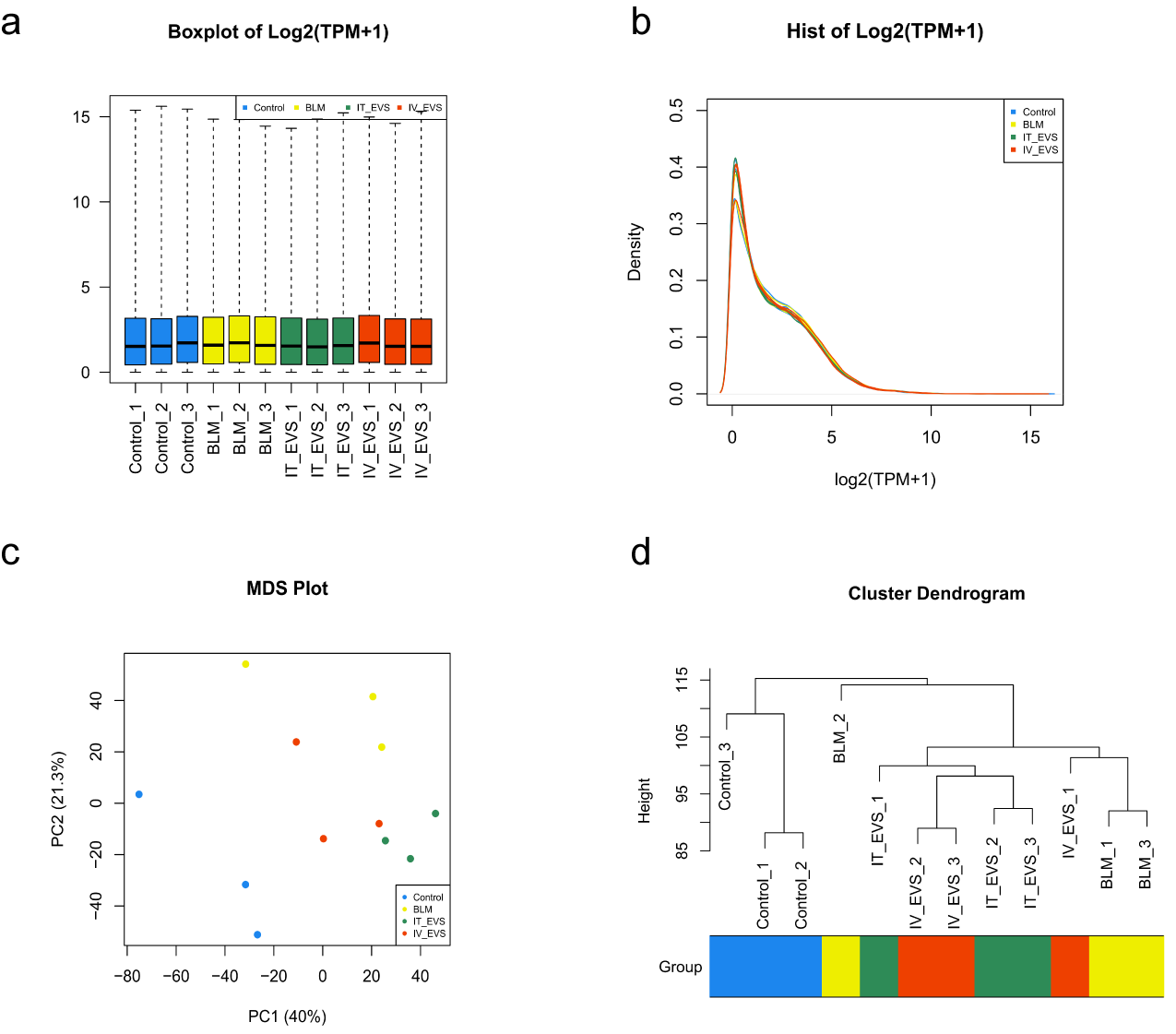

Supplement: Supplementary file 2 — Additional file 2: Figure S2. Evaluation of the RNA-seq data quality (a and b) Boxplot and expression quantity distribution density diagram among the four groups. (c and d) PCA and cluster analysis show that BLM-treated mice had different RNA expression profiles compared to control groups). [file 13287_2022_2839_MOESM2_ESM.tif]

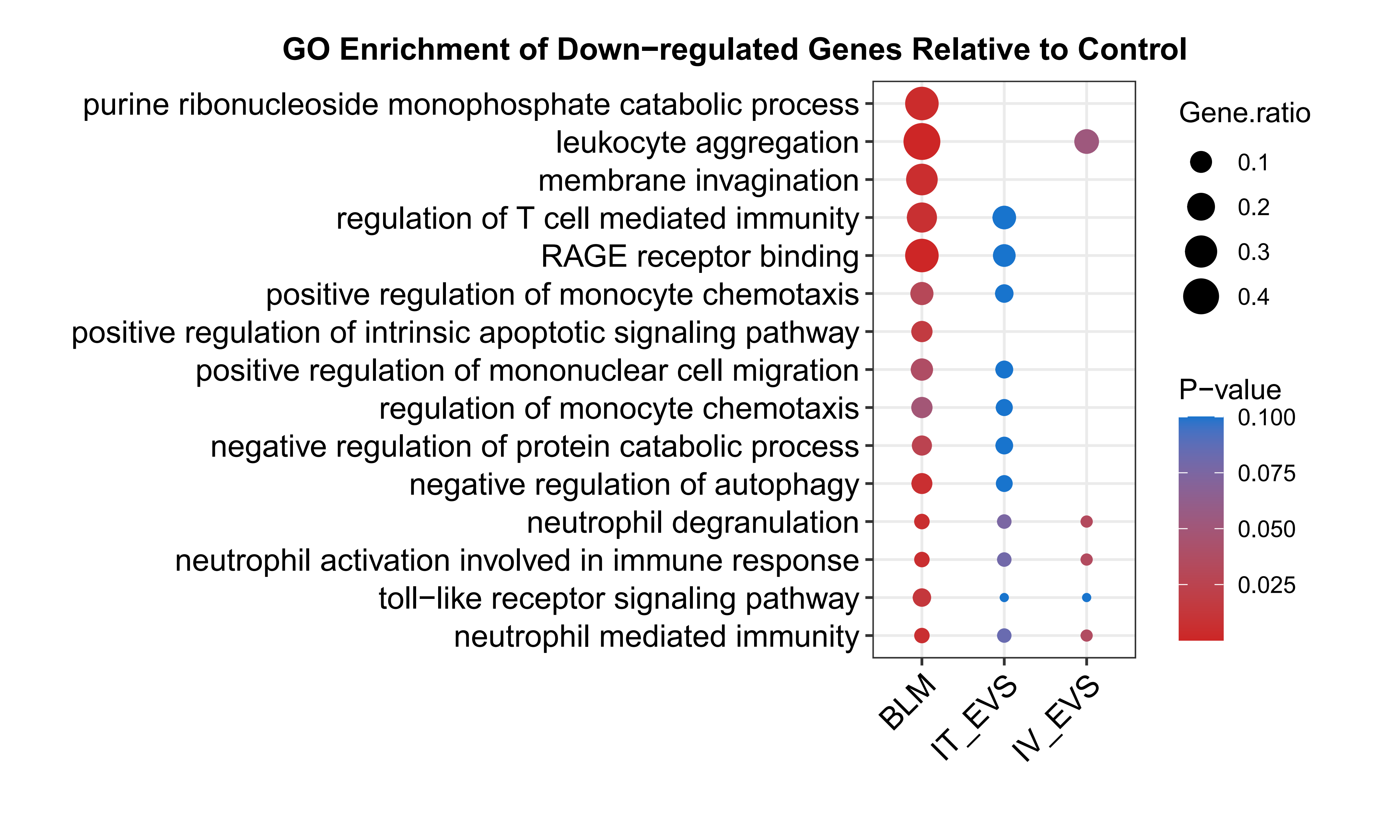

Supplement: Supplementary file 3 — Additional file 3: Figure S3. GO enrichment of down-regulated genes relative to control group. [file 13287_2022_2839_MOESM3_ESM.tif]

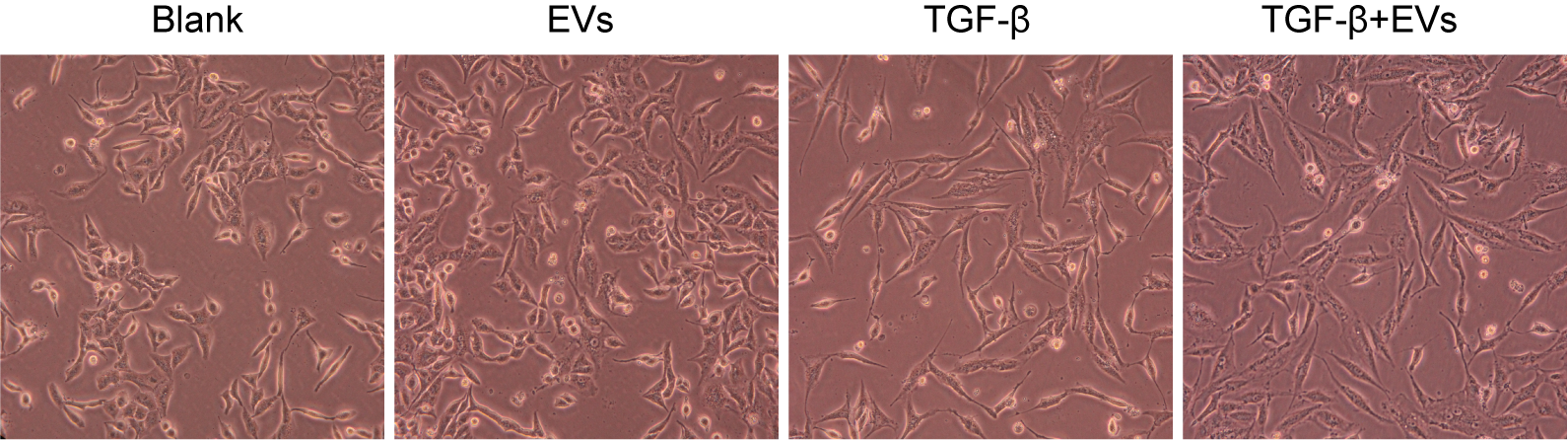

Supplement: Supplementary file 4 — Additional file 4: Figure S4. IMRC-EVs reduce the pro-fibrotic effects of TGF-β1. Representative morphology of A549 cells, with or without 2 ng/mL TGF-β1 and IMRC-EVs treatment for 48 hours. [file 13287_2022_2839_MOESM4_ESM.tif]
