# Supplementary material for: Every road leads to Rome: therapeutic effect and mechanism of the extracellular vesicles of human embryonic stem cell-derived immune and matrix regulatory cells administered to mouse models of pulmonary fibrosis through different routes
Source: Stem Cell Res Ther. 2022 Apr 12;13:163. doi: 10.1186/s13287-022-02839-7 (PMC9006546; doi:10.1186/s13287-022-02839-7)
Supplement: Supplementary file 5 — Additional file 5: Table S1. Primers used for PCR amplification. [file 13287_2022_2839_MOESM5_ESM.docx]

**Supplementary Material**

**TABLE S1 ▏List of Primers used for qRT-PCR**

| Gene name | Forward (5'-3') | Reverse (5'-3') |
| --- | --- | --- |
| H-E-cadherin | GAGAACGCATTGCCACATACAC | GAGCACCTTCCATGACAGACCC |
| H-COL1A1 | CCAAGACGAAGACATCCCACCA | CCGTTGTCGCAGACGCAGAT |
| H-GAPDH | GGAAGCTTGTCATCAATGGAAATC | TGATGACCCTTTTGGCTCCC |
