# Supplementary material for: Every road leads to Rome: therapeutic effect and mechanism of the extracellular vesicles of human embryonic stem cell-derived immune and matrix regulatory cells administered to mouse models of pulmonary fibrosis through different routes
Source: Stem Cell Res Ther. 2022 Apr 12;13:163. doi: 10.1186/s13287-022-02839-7 (PMC9006546; doi:10.1186/s13287-022-02839-7)
Supplement: Supplementary file 6 — Additional file 6: Table S2. Peak Analysis of IMRC-EVs and MRC5-EVs. [file 13287_2022_2839_MOESM6_ESM.docx]

**Supplementary Material**

**TABLE S2 ▏Peak Analysis of IMRC-EVs and MRC5-EVs**

| IMRC-EVs diameter / nm | Particles/ml | FWHM / nm | Percentage |
| --- | --- | --- | --- |
| 124.3 | 3.8E+6 | 81.0 | 99.0 |
| 26.7 | 3.3E+4 | 5.5 | 0.6 |
| 5.7 | 1.6E+4 | 0.9 | 0.1 |
| 12.0 | 5.2E+3 | 1.5 | 0.0 |
| 588.9 | 4.3E+3 | 170.9 | 0.1 |

| MRC5-EVs diameter / nm | Particles/mL | FWHM / nm | Percentage |
| --- | --- | --- | --- |
| 132.9 | 4.4E+6 | 88.2 | 98.6 |
| 33.2 | 4.6E+4 | 4.2 | 0.1 |
| 684.1 | 2.7E+4 | 153.2 | 0.1 |
| 523.9 | 2.6E+4 | 114.9 | 0.1 |
| 911.4 | 2.3E+4 | 204.3 | 0.1 |
