# Supplementary material for: Every road leads to Rome: therapeutic effect and mechanism of the extracellular vesicles of human embryonic stem cell-derived immune and matrix regulatory cells administered to mouse models of pulmonary fibrosis through different routes
Source: Stem Cell Res Ther. 2022 Apr 12;13:163. doi: 10.1186/s13287-022-02839-7 (PMC9006546; doi:10.1186/s13287-022-02839-7)
Supplement: Supplementary file 7 — Additional file 7: Table S3. Liver and kidney toxicity of IMRC-EVs in mice. [file 13287_2022_2839_MOESM7_ESM.doc]

**TABLE S3 ▏****Liver and kidney toxicity of IMRC-EVs in mice**

| Group | ALT  (IU/L) | AST  (IU/L) | ALP  (IU/L) | CK  (IU/L) | LDH  (IU/L) | TBIL  (μmol/L) |
| --- | --- | --- | --- | --- | --- | --- |
| Control | 39.60±3.62 | 165.6±43.37 | 281.50±26.61 | 513.80±162.19 | 629.80±42.21 | 6.26±2.11 |
| Control+200 μgEV | 37.50±2.18 | 195.00±40.74 | 302.00±16.67 | 678.80±133.66 | 760.60±45.96 | 6.92±0.62 |
| Control+1000 μgEV | 40.4±2.47 | 177.70±35.05 | 293.80±19.54 | 687.40±165.83 | 717.10±73.36 | 6.55±0.63 |
| Group | UREA  (mmol/L) | CREA  (μmol/L) | GLU  (mmol/L) | TG  (mmol/L) | TP  (g/L) | ALB  (g/L) |
| Control | 10.45±0.46 | 31.10±0.99 | 9.00±1.32 | 0.75±0.04 | 45.50±1.35 | 36.10±1.99 |
| Control+200 μgEV | 9.21±0.60 | 31.70±1.35 | 10.27±0.22 | 0.87±0.05 | 48.90±0.98 | 38.19±0.92 |
| Control+1000 μgEV | 9.58±0.42 | 31.30±1.59 | 10.46±0.53 | 0.82±0.05 | 46.20±1.30 | 37.70±0.79 |

Abbreviations: ALT, alanine aminotransferase; AST, aspartate aminotransferase; ALP, alkaline phosphatase; LDH,lactic dehydrogenase; CK, creatine kinase; TBIL, total bilirubin; CREA, creatinine; GLU, glucose; TG, triglyceride; TP, total protein; ALB, albumin.
