# Supplementary material for: Every road leads to Rome: therapeutic effect and mechanism of the extracellular vesicles of human embryonic stem cell-derived immune and matrix regulatory cells administered to mouse models of pulmonary fibrosis through different routes
Source: Stem Cell Res Ther. 2022 Apr 12;13:163. doi: 10.1186/s13287-022-02839-7 (PMC9006546; doi:10.1186/s13287-022-02839-7)
Supplement: Supplementary file 8 — Additional file 8: Table S4. List of differential Gene Expression in mouse lung tissue. [file 13287_2022_2839_MOESM8_ESM.docx]

**TABLE S4 ▏List of differential Gene Expression in mouse lung tissue**

| **Gene name** | **Control** | **BLM** | **IT_EVS** | **IV_EVS** | **ID** | **logFC.BLM_vs_Control** | **P.Value.BLM_vs_Control** | **Gene description** |
| --- | --- | --- | --- | --- | --- | --- | --- | --- |
| Apbb1 | 1.593333 | 0 | 3.84 | 1.103333 | ENSMUST00000211614 | -1.36858 | 2.71E-05 | Amyloid beta (A4) precursor protein-binding, family B, member 1 |
| Wasf2 | 9.613333 | 0.723333 | 12.13 | 3.59 | ENSMUST00000105912 | -2.63741 | 0.000706 | WAS protein family, member 2 |
| Dab2ip | 3.71 | 0.343333 | 4.666667 | 2.05 | ENSMUST00000112987 | -1.78877 | 0.00745 | Disabled 2 interacting protein |
| Hmgb1 | 74.82 | 27.30333 | 94.05 | 53.93 | ENSMUST00000138553 | -1.41409 | 0.010452 | High mobility group box 1 |
| Ampd3 | 9.126667 | 2.713333 | 5.52 | 4.983333 | ENSMUST00000213373 | -1.46231 | 0.000709 | Adenosine monophosphate deaminase 3 |
| Slc15a4 | 2.556667 | 0.18 | 1.846667 | 0.353333 | ENSMUST00000198486 | -1.56346 | 0.002742 | Solute carrier family 15, member 4 |
| Ap3s1 | 4.49 | 0 | 2.573333 | 2.193333 | ENSMUST00000224622 | -2.33217 | 0.000861 | Adaptor-related protein complex 3, sigma 1 subunit |
| Il1b | 32.77667 | 14.19333 | 13.93 | 16.14 | ENSMUST00000028881 | -1.14011 | 0.000429 | Interleukin 1 beta |
| Snrnp70 | 16.06333 | 6.503333 | 6.323333 | 8.41 | ENSMUST00000211121 | -1.17127 | 0.001666 | Small nuclear ribonucleoprotein 70 (U1) |
| Mmp9 | 63.53667 | 13.67 | 25.50667 | 24.45333 | ENSMUST00000017881 | -2.01951 | 0.004019 | Matrix metallopeptidase 9 |
| Rasgrp4 | 5.546667 | 1.8 | 1.74 | 2.31 | ENSMUST00000159975 | -1.20798 | 0.000431 | RAS guanyl releasing protein 4 |
| Pilra | 3.076667 | 0.73 | 0.856667 | 1.063333 | ENSMUST00000110980 | -1.21648 | 0.000631 | Paired immunoglobin-like type 2 receptor alpha |
| Fscn1 | 1.07 | 3.836667 | 5.63 | 4.636667 | ENSMUST00000129306 | 1.340241 | 0.018277 | Fascin actin-bundling protein 1 |
| Sgk1 | 2.63 | 15.89333 | 20.1 | 14.42333 | ENSMUST00000150089 | 2.204155 | 0.000441 | Serum/glucocorticoid regulated kinase 1 |
| Ccl2 | 0.666667 | 2.606667 | 3.43 | 2.366667 | ENSMUST00000000193 | 1.116679 | 0.000596 | Chemokine (C-C motif) ligand 2 |
| Ntrk2 | 0.64 | 3.393333 | 4.14 | 1.646667 | ENSMUST00000224402 | 1.449142 | 0.001081 | Neurotrophic tyrosine kinase, receptor, type 2 |
| Col18a1 | 8.813333 | 25.41 | 18.25333 | 11.19333 | ENSMUST00000081654 | 1.329982 | 0.011142 | Collagen, type XVIII, alpha 1 |
| Pex2 | 0.923333 | 4.223333 | 2.763333 | 2.78 | ENSMUST00000191916 | 1.527617 | 0.007656 | Peroxisomal biogenesis factor 2 |
| Sytl5 | 0.666667 | 2.613333 | 1.643333 | 1.693333 | ENSMUST00000086165 | 1.110881 | 0.000176 | Synaptotagmin-like 5 |
| Col6a3 | 19.60333 | 61.76667 | 43.52333 | 27.29667 | ENSMUST00000097653 | 1.639634 | 0.000846 | Collagen, type VI, alpha 3 |
| Mfap5 | 17.40333 | 49.25 | 33.15667 | 24.81 | ENSMUST00000118626 | 1.320699 | 0.014298 | Microfibrillar associated protein 5 |
| Msr1 | 1.99 | 8.683333 | 6.013333 | 4.413333 | ENSMUST00000170091 | 1.667511 | 0.000173 | Macrophage scavenger receptor 1 |
| Lhfpl2 | 1.686667 | 5.126667 | 3.423333 | 2.55 | ENSMUST00000054274 | 1.229475 | 0.005608 | Lipoma HMGIC fusion partner-like 2 |
| Sox17 | 2.096667 | 7.673333 | 3.886667 | 5.426667 | ENSMUST00000195555 | 1.429019 | 0.001728 | SRY (sex determining region Y)-box 17 |
| Rapgef1 | 2.046667 | 5.793333 | 2.876667 | 3.87 | ENSMUST00000147488 | 1.276514 | 0.024349 | Rap guanine nucleotide exchange factor (GEF) 1 |
| Pard3 | 0.273333 | 2.16 | 0.923333 | 1.623333 | ENSMUST00000162907 | 1.239658 | 0.013295 | Par-3 family cell polarity regulator |
| Igf1 | 3.046667 | 27.77667 | 19.97667 | 8.803333 | ENSMUST00000095360 | 2.793432 | 0.000251 | Insulin-like growth factor 1 |
| Plec | 1.713333 | 4.926667 | 2.466667 | 3.21 | ENSMUST00000166428 | 1.128781 | 4.34E-05 | Plectin |
| Fn1 | 46.4 | 201.8567 | 131.44 | 85.50333 | ENSMUST00000190780 | 2.092557 | 0.000296 | Fibronectin 1 |
| Dynll1 | 42.00667 | 91.43333 | 28.86333 | 57.36333 | ENSMUST00000112090 | 1.099435 | 0.000343 | Dynein light chain LC8-type 1 |
| Sulf2 | 1.236667 | 3.946667 | 1.923333 | 2.313333 | ENSMUST00000139266 | 1.186411 | 0.004119 | Sulfatase 2 |
| Itga7 | 1.14 | 3.61 | 2.18 | 1.326667 | ENSMUST00000218290 | 1.092598 | 0.000506 | Integrin alpha 7 |
| Ap2m1 | 2.62 | 10.36667 | 6.03 | 4.95 | ENSMUST00000232001 | 2.410984 | 0.036643 | Adaptor-related protein complex 2, mu 1 subunit |
| Sdcbp | 10.83333 | 25.55 | 8.823333 | 15.22667 | ENSMUST00000175769 | 1.158049 | 0.000262 | Syndecan binding protein |
| Llgl2 | 0.89 | 3.223333 | 1.58 | 1.84 | ENSMUST00000128826 | 1.148399 | 0.001463 | LLGL2 scribble cell polarity complex component |
| Postn | 3.173333 | 12.89 | 7.716667 | 4.996667 | ENSMUST00000073012 | 1.598197 | 0.009072 | Periostin, osteoblast specific factor |
| Mfap4 | 4.48 | 9.72 | 4.01 | 4.776667 | ENSMUST00000040522 | 1.061774 | 0.024227 | Microfibrillar-associated protein 4 |
| Nrcam | 1.663333 | 6.47 | 3.71 | 2.47 | ENSMUST00000110748 | 1.534588 | 0.000998 | Neuronal cell adhesion molecule |
| Pafah1b2 | 0.596667 | 4.046667 | 2.076667 | 1.14 | ENSMUST00000213853 | 1.635538 | 0.039073 | Platelet-activating factor acetylhydrolase, isoform 1b, subunit 2 |
| Atp13a3 | 1.096667 | 6.393333 | 2.076667 | 3.1 | ENSMUST00000229503 | 2.078596 | 0.022531 | ATPase type 13A3 |
| Plcb1 | 0.793333 | 3.406667 | 0.926667 | 1.483333 | ENSMUST00000110116 | 1.323701 | 0.000719 | Phospholipase C, beta 1 |
| Anpep | 1.946667 | 6.753333 | 1.913333 | 1.286667 | ENSMUST00000205502 | 1.418089 | 0.024137 | Alanyl (membrane) aminopeptidase |
| Rap1a | 1.556667 | 11.21333 | 0 | 4.766667 | ENSMUST00000197094 | 2.671169 | 0.012958 | RAS-related protein 1a |
| Kat5 | 0.116667 | 2.746667 | 0.626667 | 0.793333 | ENSMUST00000236264 | 1.728787 | 0.000308 | K(lysine) acetyltransferase 5 |
| Arf4 | 1.79 | 13.44667 | 1.56 | 0.55 | ENSMUST00000112318 | 2.849815 | 0.013127 | ADP-ribosylation factor 4 |
| Creg1 | 1.223333 | 22.87333 | 0 | 5.386667 | ENSMUST00000040298 | 3.507274 | 0.007657 | Cellular repressor of E1A-stimulated genes 1 |
